# Imaging skins: stretchable Gd2O2S:Tb X-ray detectors for image-guided surgery

**DOI:** 10.1007/s11548-026-03681-5

**Published:** 2026-05-11

**Authors:** Rozenn Raffaut, Robert Moss, Agostino Stilli, Danail Stoyanov

**Affiliations:** 1 Hawkes Institute, University College London, London, WC1E 6BT UK; 2 Department of Medical Physics and Biomedical Engineering, University College London, London, WC1E 6BT UK; 3 Department of Computer Science, University College London, London, United Kingdom

**Keywords:** Detector characterisation, Image quality, Inorganic scintillator, Stretchable sensor, X-ray detector

## Abstract

****Purpose**:**

With the growing demands for diverse X-ray imaging applications, there is a need for the development of flexible X-ray detectors to enable conformity to curved surfaces, improving spatial resolution, reducing X-ray exposure, and extending the potential of X-ray imaging to fields such as minimally invasive surgery, which would not be achievable using conventional rigid flat-panel detectors.

****Methods**:**

In this paper, we fabricate imaging skins—flexible and stretchable Gd2O2S:Tb-elastomer scintillator films—and integrate them into an indirect X-ray imaging system. We characterise X-ray imaging performance and mechanical properties of the imaging skins, investigating the impact of varying film fabrication parameters such as thickness and concentration.

****Results**:**

The results demonstrate the clinical suitability of the imaging skin system, achieving 1.05 to 2.18 lp/mm at 10% contrast despite the low X-ray energy used, which is comparable to clinical CT. Additionally, the high stretchability (255%) from the elastomer component was maintained in imaging skins with up to 50% Gd2O2S:Tb loading.

****Conclusion**:**

This work provides a basis for the integration of imaging skins in clinical settings, where they have the potential to be used for real-time intraoperative image guidance. Future work will investigate dynamic and tomographic imaging methods, determine effective deployment strategies of the imaging skins onto organ surfaces, and investigate the effects of imaging skin deformation on image quality.

## Introduction

Flat-panel indirect X-ray detectors, consisting of a scintillator layer paired with a photodetector panel, are a crucial component of most modern X-ray systems [1]. However, several X-ray imaging applications have emerged that require large-area flexible detectors that can conform to irregular shapes. This has led to advances in flexible X-ray scintillator development in recent years, with potential to reduce radiation exposure, improve resolution, miniaturise imaging systems, and provide more user-friendly deformable alternatives to rigid flat-panel detectors [2].

In surgery, soft robotic devices have been developed to overcome the limitations of rigid-body robots [3]. For example, the PAF rail is a flexible surgical instrument that enables track-guided intraoperative ultrasound image guidance for robotic-assisted partial nephrectomy by complying to organ curvature [4] and the STIFF-FLOP manipulator addresses the lack of dexterity and instrument localisation in minimally invasive procedures using a soft robotic arm with tunable stiffness and integrated flexible optic-based proprioceptive sensing [5, 6].

**Fig. 1:**
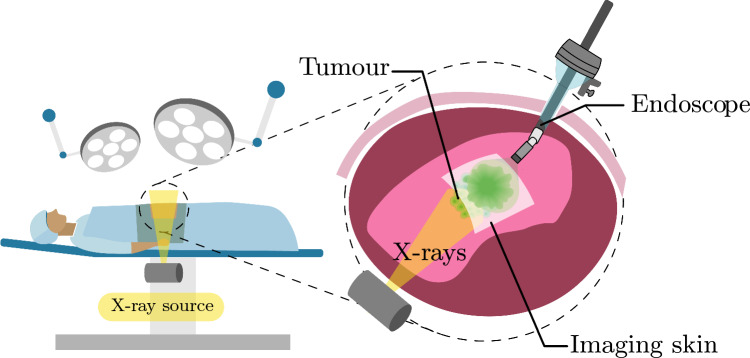
Imaging skins concept. The imaging skin is inserted through a trocar port and placed on the surface of the organ of interest during minimally invasive tumour resection surgery. The X-ray source is activated, and the visible scintillation light emitted by the imaging skin is captured by the surgical endoscope, achieving indirect X-ray imaging of the tumour. Graphic reproduced from [9] under CC BY 4.0 (To view a copy of this license, visit http://creativecommons.org/licenses/by/4.0/)

*Imaging skins*, named for their conceptual similarity to e-skins, are flexible Gd2O2S:Tb (GOS:Tb)-elastomer composite films that aim to provide X-ray image guidance for minimally invasive tumour resection surgery [7, 8]. As illustrated in Fig. 1, the scintillator component enables the surgeon to visualise the internal structures of organs using the existing surgical endoscope system to detect the emitted visible scintillation light from the imaging skin, which is placed directly onto the organ of interest. The elastomer component allows the imaging skins to easily deform to fit in a trocar port during surgical deployment and to comply to curved organ surfaces. Imaging skins have the potential to improve surgical outcomes by reducing the occurrence of positive surgical margins [7, 9]. To achieve this, the spatial resolution of the imaging skin system should match or exceed that of current intraoperative or preoperative tumour imaging methods while maintaining high contrast between the tumour and healthy tissue.

Existing tumour localisation methods include real-time fluorescence imaging, which uses fluorescent dyes such as indocyanine green (ICG) to visualise tumours during surgery without requiring ionising radiation. Spatial resolution is poor and visualisation is restricted to a few millimetres from the tissue surface due to the infrared or visible light fluorescence being scattered or absorbed by normal tissue [10]. Preoperative imaging such as computed tomography (CT) or magnetic resonance imaging (MRI) are also used to guide surgical procedures, where the surgeon can spatially map the knowledge gained through these images to the surgical scene observed [10]. Conventional CT scanners generally achieve submillimetre resolutions or poorer, with voxel sizes over 0.1 mm [11] and resolutions over 2 lp/mm [12], and clinical MRI scanners can achieve pixel sizes from 0.28 to 1.35 mm depending on the scanning sequence used [13].

Furthermore, to be used as real-time on-organ X-ray imaging detectors, imaging skins should retain flexible and stretchable material properties from their elastomer component to remain compliant to organ surfaces, even during breathing motion. High tensile strength and durability are essential to ensure the skin does not get damaged during deployment or handling. For flexible scintillating films, the addition of scintillator powder to a silicone elastomer has been found to both increase or decrease tensile strength, depending on the materials and phosphor concentrations used [14].

While there are some examples of flexible scintillator films in the literature [15, 16], few studies have investigated the interactions between their fabrication parameters and X-ray image quality metrics. The effects of varying fabrication technique on the mechanical properties of flexible scintillator films is rarely tested, and has not been investigated for GOS:Tb-elastomer imaging skins specifically [14].

This work investigated the effects of film thickness and phosphor concentration on X-ray imaging performance. The mechanical properties under tension of samples with varying phosphor-elastomer ratios were also examined, and compared with the unmodified silicone elastomer.

## Methods

### Materials

The GOS:Tb phosphor (25 µm median powder size) was purchased from Phosphor Technology Ltd., Stevenage, UK. The polydimethylsiloxane (PDMS) two-part elastomer, Sylgard 186, was purchased from Dow Inc., Midland, MI, USA.

### Imaging skin fabrication

The Sylgard 186 elastomer was prepared using the recommended Part A to Part B ratio of 10:1 by mass. The elastomer was then combined with GOS:Tb powder using GOS:Tb concentrations of 33, 50, and 67% by mass, mixed manually for 5 min, then de-gassed in a vacuum chamber for 30 min to remove air bubbles.

Imaging skins were cast from the phosphor-elastomer mixtures using custom manual film applicators with measured gap sizes of 400, 600, and 900 µm. Actual film thicknesses are assumed to be half of applicator gap size due to surface tension [17], resulting in film thicknesses of 200, 300, and 450 µm.

The range of film thicknesses used was selected following work by [9], which suggested that the optimal imaging skin thickness to maximise scintillation intensity was below 500 µm.

### Tensile testing

Four dog bone-shaped tensile test specimens were prepared using GOS:Tb concentrations 0, 33, 50, and 67% by mass.

The phosphor powder and elastomer were combined according to the desired GOS:Tb concentration and mixed manually for 5 min, then de-gassed in a vacuum chamber for 30 min. This mixture was poured into a 3D-printed mould and de-gassed in the mould for an additional five minutes to remove air bubbles. They were then left to cure and stabilise their material properties at room temperature for at least 15 days to reduce potential age-related differences in material properties between specimens [18].

The specimens had a narrow portion width of 6 mm, a narrow portion length of 33 mm, end widths of 19 mm, and a thickness of 3.2 mm, compliant with the ASTM D638 IV standard [19].

Uniaxial tension tests were performed using an INSTRON 5969 universal testing machine (Illinois Tool Works Inc., Glenview, IL, USA), providing load–displacement data.

### X-Ray imaging setup

**Fig. 2:**
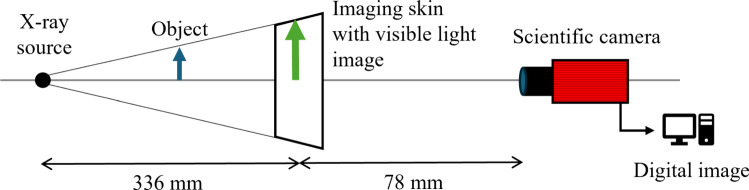
X-ray imaging experimental setup

As imaging skins are indirect X-ray detectors, they first convert absorbed X-rays into visible scintillation light photons which are then captured using a photodetector. Figure 2 shows the custom indirect X-ray imaging setup used with the imaging skins.

The X-ray source used was from a microCT system manufactured by Nikon X-Tek Systems Ltd. (Nikon Metrology UK Ltd., Derby, UK), using a tungsten target operating at outputs of up to 125 kV and 1.0 mA. No filter was used. Low X-ray tube settings of 50 kVp voltage and 0.1 mA were selected to demonstrate that the imaging skin system had high sensitivity to X-rays.

The CS505MU Kiralux Compact Scientific Digital Camera (Thorlabs Inc., Newton, NJ, USA), was used to acquire images of the scintillation light emitted by the imaging skins. It is a high-resolution (5.0 MP) monochrome CMOS camera, with parameters that can be fixed to ensure consistency between experiments. Camera settings were chosen to maximise image intensity and are listed in Table 1, alongside the imaging system geometry.

**Table 1:** Camera settings and system geometry

Setting	Value
Frames averaged per image	10
Exposure (s)	1.0$^{\text {a}}$
Gain (dB)	30
Effective image pixel pitch (µm)	23.15
Source-to-camera distance (m)	0.414
Imaging skin-to-camera distance (m)	0.078

$^{\text {a}}$An exposure value of 1.5 s was used for the 200 µm, 33% imaging skin due to lower scintillation intensity

### X-Ray imaging performance evaluation

For each of the nine imaging skins tested, 10 flat-field images without any phantom and 10 edge response images were taken. These used a 0.5-mm-thick aluminium edge phantom placed as close as possible to the imaging skin to avoid artificially improving spatial resolution through magnification. Stacks of 10 dark-field images were taken for both exposure settings used (1.0 and 1.5 s).

The edge images were preprocessed according to (1), adapted from [20], which corrects for photodetector offset and non-uniform flat-field from the imaging skin and x-ray beam. Median averaging was used due to the presence of high pixel values from electronic noise and from transmitted X-ray photons directly stimulating the camera photodetector.

1 $$\begin{aligned} I_\text {corrected} = m \times \frac{\overline{I_\text {edge}}-\overline{I_\text {dark}}}{\overline{I_\text {flat}}-\overline{I_\text {dark}}}; \quad m = \sum _{i,j}^{N_\text {pixels}}\frac{(\overline{I_\text {flat}}-\overline{I_\text {dark}})}{N_\text {pixels}} \end{aligned}$$

The edge-based method specified in the IEC 62220 standard for digital X-ray imaging devices was used to characterise spatial resolution, resulting in the edge spatial frequency response (e-SFR), which is a measurement of the modulation transfer frequency (MTF) [21].

Noise power spectra (NPS), which measure the power (squared amplitude) of noise as a function of spatial frequency, were computed using the flat-field images by following the periodogram method recommended in the IEC 62220 Standard [21] and outlined in [22]. Noise-equivalent quanta (NEQ) were then calculated using (2), where the large-area signal is the average pixel value over a large area of the image, normalising NPS amplitudes [23]. NEQ represents the effective number of photons that make up an image, and is proportional to the signal-to-noise ratio squared.

2 $$\begin{aligned} \text {NEQ}(f) = \frac{(\text {large area signal})^2(\text {MTF}(f))^2}{\text {NPS}(f)} \end{aligned}$$

## Results and discussion

### Imaging skins and mechanical properties

**Fig. 3:**
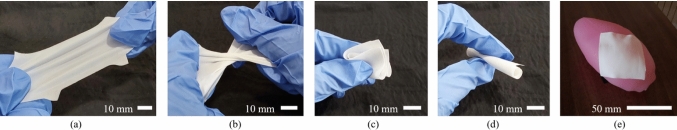
Imaging skin with 300 µm thickness and 50% GOS:Tb concentration **a** stretching, **b** twisting, **c** folding, **d** rolling, and **e** passively complying to curvature, demonstrating its flexibility

Figure 3 shows the fabricated imaging skins, demonstrating their flexibility and stretchability. These soft sensor properties can be used to introduce the films into laparoscopic surgical fields without damaging them and to take X-ray images while conforming to dynamic and irregular organ surfaces.

Dynamic gauge length values, which are necessary for calculating engineering stress and strain, were not available as no extensometer was used. Stress and strain values were therefore estimated by scaling the Sylgard 186 elastomer (0% GOS:Tb) curve to the 255% elongation at break and 2.1 MPa tensile strength values reported by the manufacturer. This estimation method assumed that any potential elongation outside of the gauge area and slipping at the grips could be compensated by constant multiplicative factors.

**Fig. 4:**
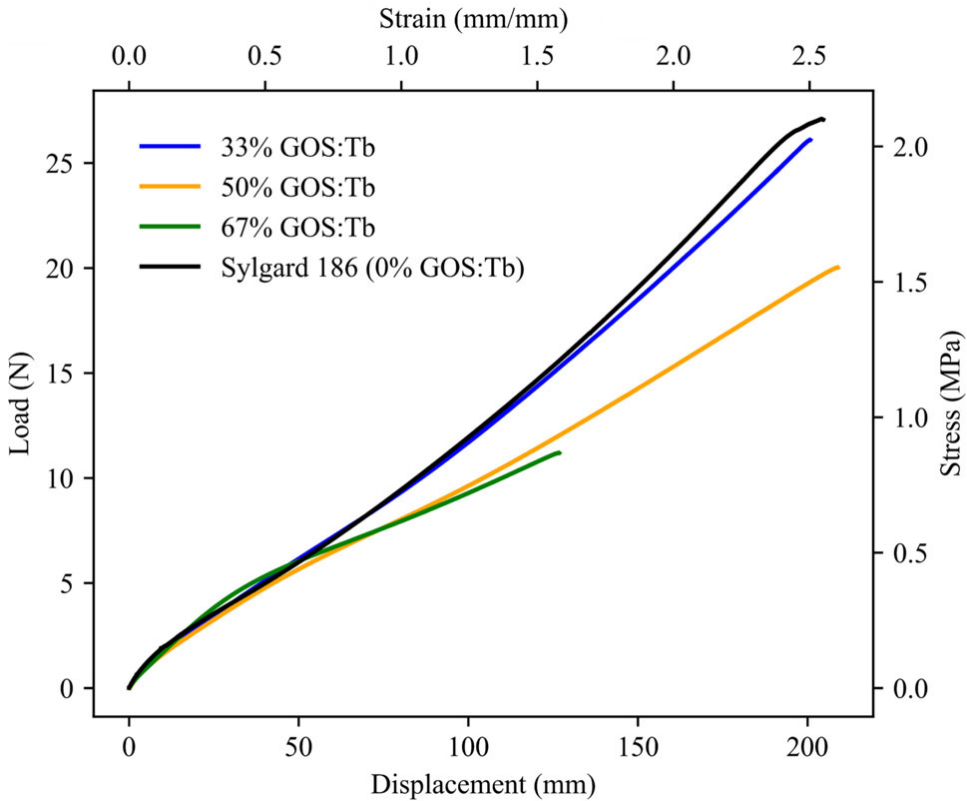
Uniaxial tension test load–displacement curves and estimated stress–strain values for four GOS:Tb-Sylgard 186 specimens with varying phosphor concentrations

Figure 4 shows measured load–displacement curves for the samples tested under uniaxial tension with estimated stress–strain values on the secondary axes. The estimated ultimate tensile strength, elongation at break, and Young’s modulus values were calculated using the estimated stress–strain curves. These are presented in Table 2.

The results show that ultimate tensile strength decreases with GOS:Tb concentration, with the largest change being between 33% and 50% GOS:Tb, and that high elongation at break (>250%) is maintained up until 50% GOS:Tb concentration. The 67% GOS:Tb specimen displays a reduced elongation at break of 158%, although this is still higher than that of the more commonly used Sylgard 184 elastomer which achieves 100% elongation at break [24].

**Table 2:** Estimated ultimate tensile strength (UTS), elongation at break, and Young’s modulus calculated at 2% strain (E) for four specimens with varying phosphor concentrations

GOS:Tb %	UTS (MPa)	Maximum elongation %	E (MPa)
0	2.10$^{\text {a}}$	255$^{\text {a}}$	2.08
33	2.02	250	1.74
50	1.55	260	0.25
67	0.87	158	1.60

$^{\text {a}}$These values were set to those reported by the manufacturer

### X-Ray imaging performance evaluation

The resolution of the imaging skin system ranged from 1.05 to 2.18 lp/mm at 10% of the e-SFR (corresponding to 0.95–0.465 mm resolution), where SFR10 is used as the limiting resolution of the system [25]. These values are comparable to the standard spatial resolutions required for medical CT scans (2 lp/mm) despite the low X-ray current and voltage settings [12].

**Fig. 5:**
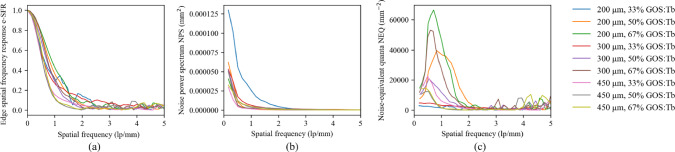
X-ray imaging performance of the nine tested imaging skins in terms of **a** e-SFR, **b** NPS, and **c** NEQ

Figure 5 compares the X-ray imaging performances for the nine imaging skins tested using the e-SFR, NPS, and NEQ. It should be noted that the NPS(f=0) and NEQ(f=0) could not be accurately estimated due to the influence of background trending artefacts on the NPS curve at low spatial frequency [22, 23].

The 200 um, 33% GOS:Tb imaging skin had consistently higher levels of noise compared to the other imaging skins, likely due to its low scintillation intensity despite the increased exposure time used in image acquisition. None of the curves showed white-spectrum noise; instead, the elevated low-spatial-frequency noise component suggests high sensitivity to fixed-pattern noise from X-ray beam non-uniformity and imaging skin phosphor distribution non-uniformity compared to high-spatial-frequency electronic noise.

The NEQ spectrum peaks can be used to compare imaging skin efficiencies: the 200 um, 67% GOS:Tb imaging skin achieves the highest NEQ due to its wide MTF curve and low noise and the 200 um, 33% GOS:Tb imaging skin achieves the worst NEQ due to its high levels of noise.

**Fig. 6:**
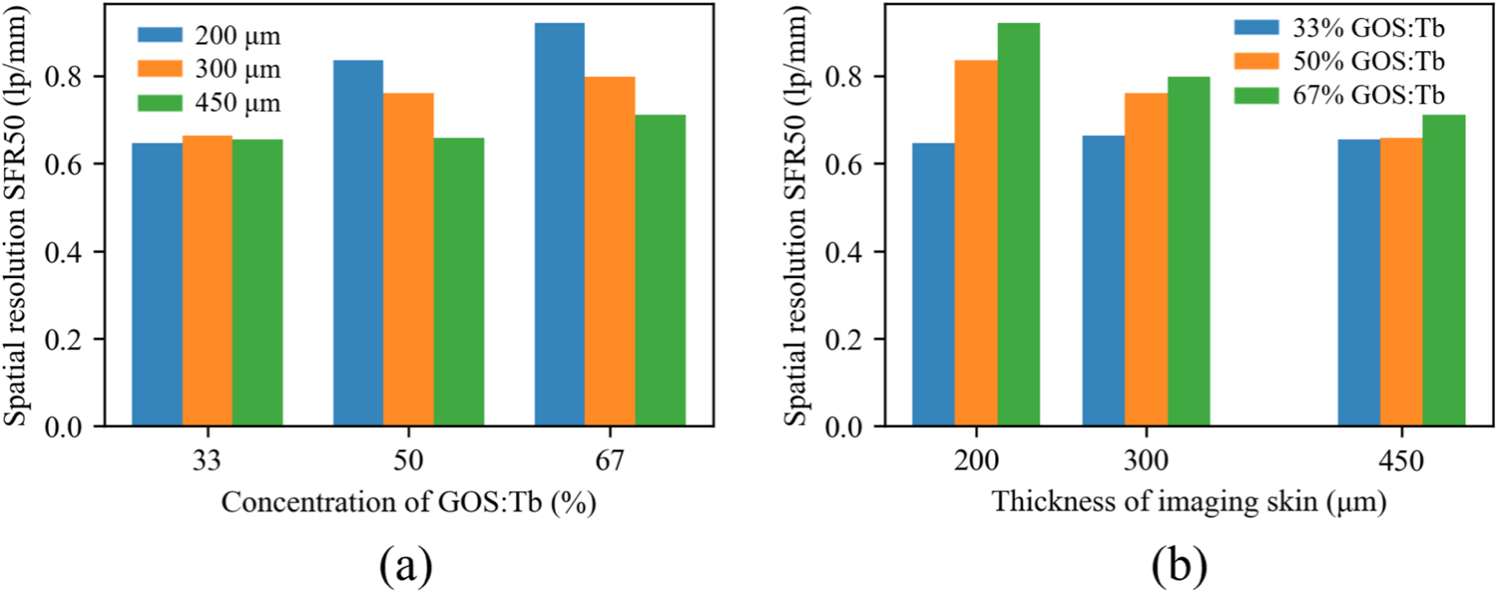
Effects of **a** GOS:Tb concentration and **b** film thickness on SFR50

Figure 6 shows the effect of imaging skin thickness and concentration on e-SFR. SFR50 is the spatial frequency level at 50% of the maximum e-SFR, and is used to quantify the width of the e-SFR curve.

SFR50 increases with concentration when film thickness is kept constant, with the slope of this effect decreasing with thicker films. SFR50 decreases with increasing film thickness for the 50% GOS:Tb films and the 67% GOS:Tb films, but not for the 33% GOS:Tb films. While the number of combinations of film fabrication parameters tested was small ($\text {n}=9$), these results suggest that increasing phosphor concentration brings the most improvement in resolution for thinner films, and that increasing thickness negatively affects the resolution of films with over 50% GOS:Tb concentration. This leads to the thinnest, highest concentration imaging skin tested (200 µm, 67% GOS:Tb) achieving the best resolution.

While other flexible scintillators have achieved higher spatial resolution levels [15], especially when directly paired with a photodetector array, the imaging skins produced in this work displayed excellent flexibility and stretchability with a facile and low-cost fabrication method, using the lead-free scintillator GOS:Tb. Spatial resolution was likely limited by low scintillation intensity and scattering effects from the finite film thickness, lack of structuring, and large GOS:Tb powder particle size [26, 27].

Based on the tensile tests, a 50% GOS:Tb concentration by mass is the most preferable of the options tested based on mechanical properties, as it is the highest concentration film that maintains high stretchability from the elastomer component. Maintaining imaging skin phosphor concentrations at 50% may therefore balance image quality and mechanical properties.

### Demonstration of X-ray imaging

**Fig. 7:**
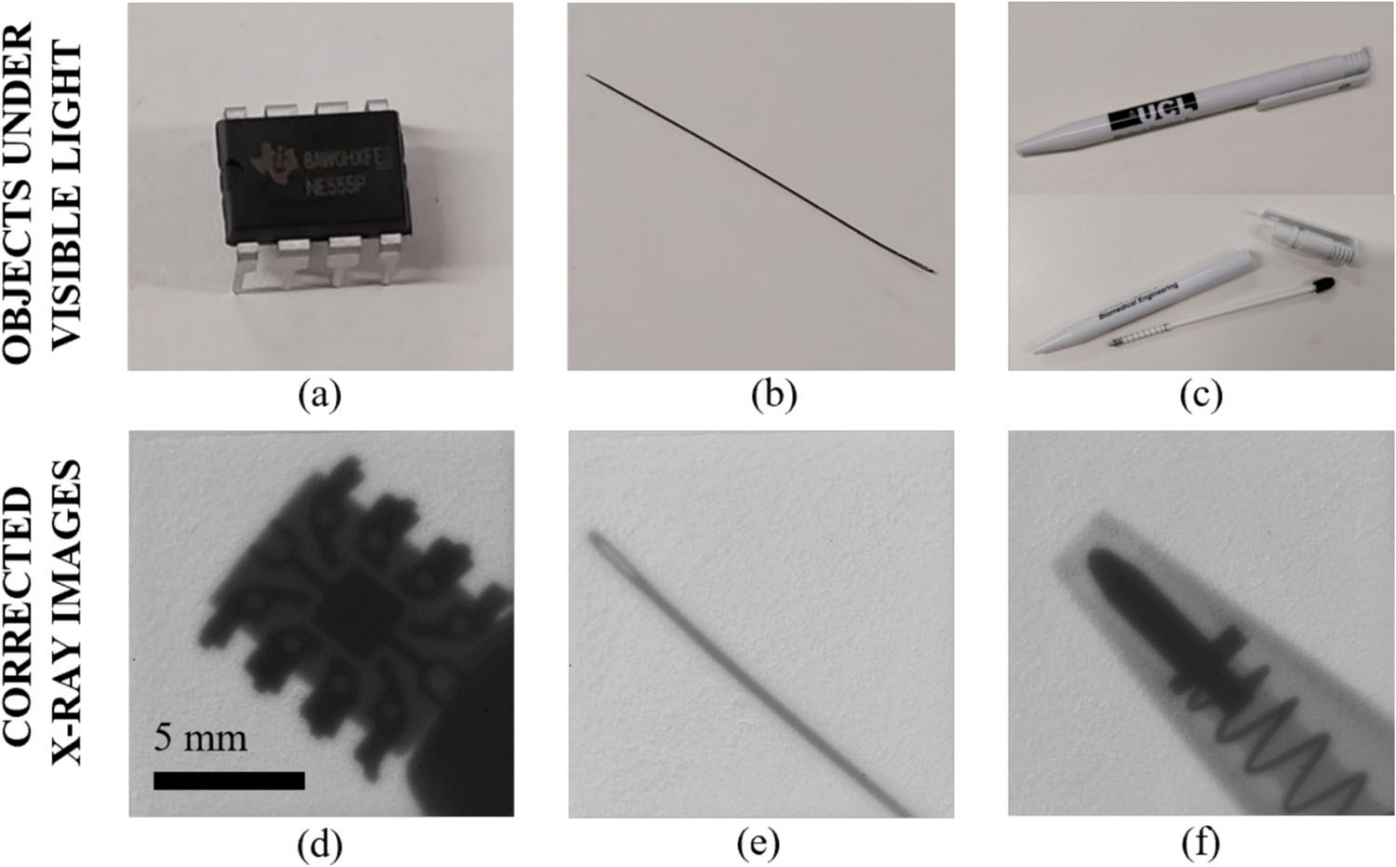
Imaging of an electronic chip, a needle, and a pen containing a spring mechanism using the imaging skin system (left to right). **a**–**c** show the objects as they appear under visible light and **d**–**f** are the X-ray images after noise corrections. The imaging skin system is able to resolve internal structures that cannot be seen under visible light

X-ray imaging was performed using the imaging skin systems to demonstrate potential for medical imaging. Figure 7 shows images of various objects taken using the 300 µm, 33% GOS:Tb imaging skin due to its high SFR10. The objects were placed 152 mm away from the source, resulting in a magnification of 2.2 and an effective resolution of 4.82 lp/mm (0.21 mm) at 10% of e-SFR. Details such as the internal structure of an electronic chip are visible in the X-ray images.

### Limitations and future work

The long exposure times, low-power X-ray beam, and median-averaged frames reduce the effect of stochastic noise, which is useful for characterising the system blur through e-SFR. However, this method of image acquisition would not be suitable for the intended application for imaging skins: real-time on-organ imaging. Scintillation light yield is expected to be linearly proportional to X-ray current [9], meaning that X-ray current could be increased appropriately to compensate for the shorter exposure times in video-rate imaging (> 24 Hz).

Measuring spatial resolution through the MTF and noise level through NPS is suitable for static 2D X-ray imaging, but does not fully characterise dynamic methods such as fluoroscopy, or tomographic methods such as CT or cone-beam CT. The MTF and NEQ metrics only measure spatial resolution within a static image without considering the temporal resolution or residual signal from previous frames in a series of images [21]. The intended use of imaging skins is real-time intraoperative surgical guidance imaging, so temporal resolution and lag correction should be considered in addition to static spatial resolution.

Mechanical deformation could be used to intentionally modulate scintillation intensity and image resolution by changing the effective film thickness and concentration [9]. For example, if a detailed image of small structures is required, the film could be stretched to a known thickness, resulting in the desired higher resolution. This could be achieved by interacting with the imaging skins using surgical grippers or by actuating the imaging skins themselves. Passive deformations of the imaging skin when conforming to organ surfaces may also produce unintentional modulations in scintillation intensity and resolution. Flat-field corrections would be less straightforward for a dynamic deformed imaging skin, as the instantaneous flat-field image would be unknown. The imaging skin and X-ray source poses would need to tracked to apply predicted corrections in real-time [28]. Further testing should be conducted to determine the effects of deformation on scintillation intensity and imaging performance.

## Conclusion

The spatial resolution and NPS of GOS:Tb-silicone imaging skins with varying fabrication parameters has been tested, achieving 1.05 to 2.18 lp/mm resolution at 10% of the e-SFR without magnification. Spatial resolution increased with concentration and decreased with film thickness. This led to the thinnest, highest concentration imaging skin tested (200 µm, 67% GOS:Tb) achieving the best resolution, although tensile testing showed that maximum elongation decreased after 50% GOS:Tb concentration. The imaging skin achieving the second-best resolution, which was 200 µm thick but contained 50% GOS:Tb, may therefore be preferable overall when mechanical properties are considered.

These results show the clinical suitability of imaging skins, as the resolutions achieved are comparable to those required in clinical CT scans (2 lp/mm) despite the low X-ray current and voltage settings used [12]. Future work is needed to extend the use of imaging skins to dynamic and 3D imaging methods, determine effective deployment strategies of the imaging skins onto organ surfaces in minimally invasive surgery, and investigate the effects of imaging skin deformation on image quality.
